# Strategies targeting hemagglutinin cocktail as a potential universal influenza vaccine

**DOI:** 10.3389/fmicb.2022.1014122

**Published:** 2022-09-29

**Authors:** Xuejie Liu, Tianyi Zhao, Liangliang Wang, Minchao Li, Caijun Sun, Yuelong Shu

**Affiliations:** ^1^School of Public Health (Shenzhen), Sun Yat-sen University, Shenzhen, China; ^2^Institute of Pathogen Biology, Chinese Academy of Medical Sciences and Peking Union Medical College, Beijing, China

**Keywords:** influenza, hemagglutinin, universal vaccine, antigen design, T-cell epitopes, CTL epitopes

## Abstract

Vaccination is the most effective means of protecting people from influenza virus infection. The effectiveness of existing vaccines is very limited due to antigenic drift of the influenza virus. Therefore, there is a requirement to develop a universal vaccine that provides broad and long-lasting protection against influenza. CD8+ T-cell response played a vital role in controlling influenza virus infection, reducing viral load, and less clinical syndrome. In this study, we optimized the HA sequences of human seasonal influenza viruses (H1N1, H3N2, Victoria, and Yamagata) by designing multivalent vaccine antigen sets using a mosaic vaccine design strategy and genetic algorithms, and designed an HA mosaic cocktail containing the most potential CTL epitopes of seasonal influenza viruses. We then tested the recombinant mosaic antigen, which has a significant number of potential T-cell epitopes. Results from genetic evolutionary analyses and 3D structural simulations demonstrated its potential to be an effective immunogen. In addition, we have modified an existing neutralizing antibody-based seasonal influenza virus vaccine to include a component that activates cross-protective T cells, which would provide an attractive strategy for improving human protection against seasonal influenza virus drift and mutation and provide an idea for the development of a rationally designed influenza vaccine targeting T lymphocyte immunity.

## Introduction

**Figure 1:**
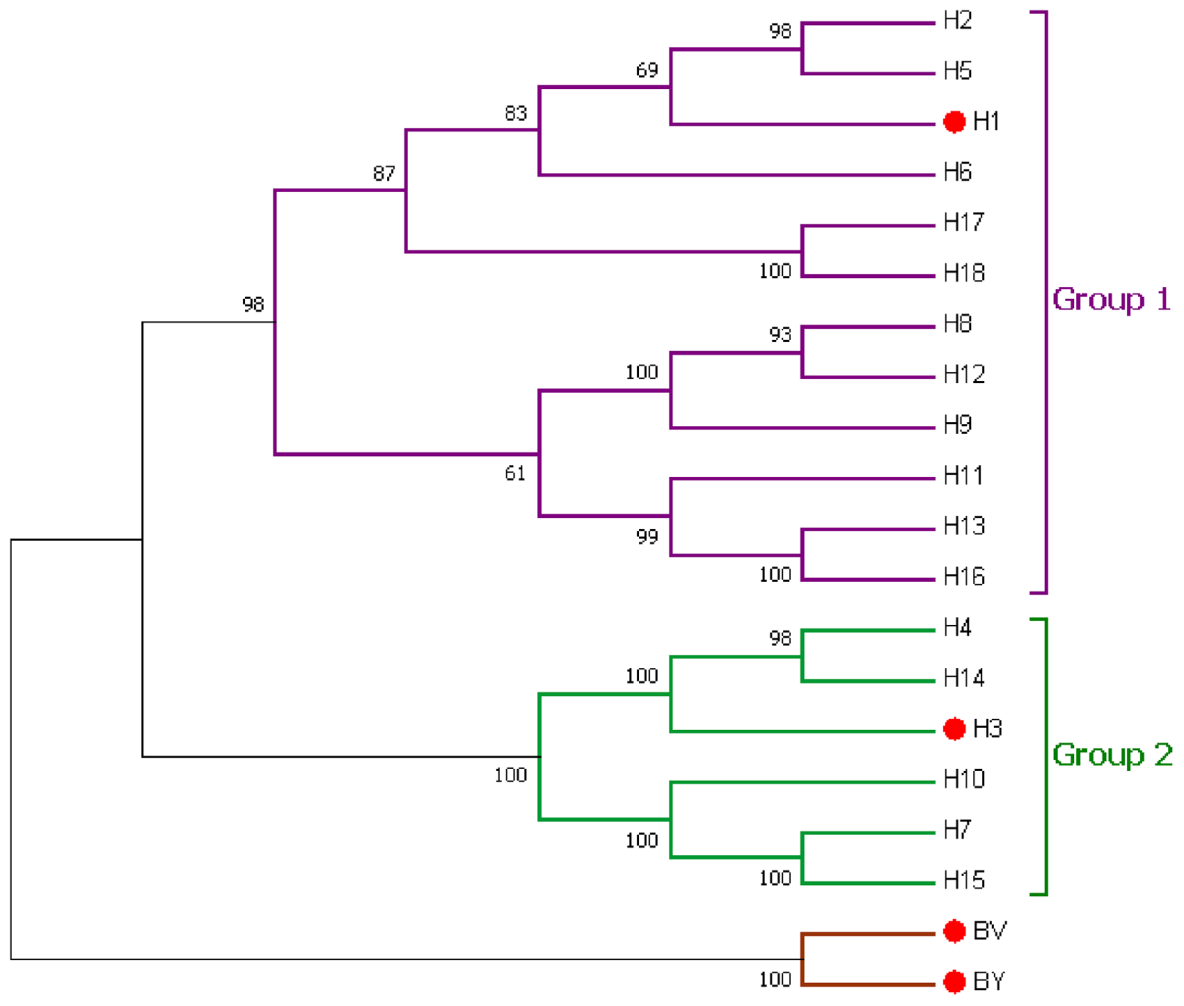
Phylogenetic relationship of the influenza A and influenza B hemagglutinin proteins. IAVs Group 1 (violet) and Group 2 (green) phylogenic groups are indicated. Seasonal influenza HA (H1, H3, BV, and BY) is marked by red dots. Representative strains for each HA subtype were aligned using ClustalW alignment and a maximum likelihood phylogenetic tree was constructed using MEGA7.

Influenza (flu) is a contagious respiratory disease that caused severe health impacts globally. There are two main types of influenza (flu) viruses circulating in the human population: Type A and B. Influenza A viruses (IAVs) are classified according to 18 HA subtypes and 11 NA subtypes, while influenza B viruses (IBVs) are classified into two lineages, Victoria and Yamagata (Hause et al., 2013; Zhang et al., 2020). The 18 HA subtypes of IAV are divided into phylogenetic groups 1 and 2 (Figure 1). H1N1, H3N2, and two types of influenza B lineages that are routinely circulating in humans (human influenza viruses) caused seasonal influenza epidemics each year. Influenza vaccination is the best intervention to prevent influenza, and reduce the risk and complications of influenza. However, the HA and NA antigens have a high mutation frequency which could cause antigenic drift and antigenic shift, leading to immune escape of influenza viruses and triggering seasonal influenza epidemics and global influenza pandemics (Taubenberger and Kash, 2010; Lopez and Legge, 2020). Statistics from the CDC show that vaccine effectiveness is 38%–60% when the vaccine strain is matched to a prevalent strain (Treanor et al., 2012; Jackson et al., 2017; Rolfes et al., 2019) and only 10%–19% when the strain is mismatched (Belongia et al., 2009; Zimmerman et al., 2016). Current influenza vaccines do not have sufficient capacity to induce broad cross-protection against antigenically drifted or transformed prevalent strains, and their effectiveness is influenced by the degree to which the vaccine strain matches the prevalent strain. Therefore, there is an urgent need to develop a strategic approach to produce a universal influenza vaccine.

Many universal influenza vaccine strategies are currently being developed worldwide. Viral surface glycoproteins, hemagglutinin (HA), and neuraminidase (NA) are the most common targets of vaccines, but their cross-protection is limited. Recent studies have focused on the HA stem region, which is more conserved than the HA head region. HA and NA targets rely on antibody responses against IAV. However, the importance of T-cell immune responses has been ignored in current influenza vaccines (Kaur et al., 2011; Kanekiyo et al., 2019; Nguyen-Contant et al., 2021). The T-cell immune response plays an essential role in controlling influenza virus infections (Jansen et al., 2019). There is also growing evidence that T-cell immunity may be the core factor for cross-protection vaccine development (Wang et al., 2007; Brown and Kelso, 2009; Cargnelutti et al., 2013). Furuya et al. (2010) adopted adoptive transfer of memory T cells from influenza virus-infected mice into normal mice that were not infected with the virus, and found that the mice were immune to challenge from different subtypes of influenza virus. Immunization with live attenuated H9N2 vaccine in chickens protects chickens from H5N2 virus challenge due to the presence of cross-memory T cells (Wang et al., 2018). In addition, the degree of protection against different subtypes of influenza was also positively correlated with the number of responding T cells (Yan et al., 2017). These studies have directly or indirectly indicated that T-cell immunity is a key consideration when designing a universal influenza vaccine. Using the mosaic vaccine method to design vaccine sequences that can contain more potential T-cell epitopes. This approach has been used in vaccine development against dengue virus, human HIV, and influenza virus, and has been able to elicit stronger cross-reactive T-cell immunity (Kamlangdee et al., 2016; Barouch et al., 2018; Hou et al., 2019).

Therefore, in this study, we used the Mosaic vaccine strategy and genetic algorithms to design a multivalent vaccine antigen set, focusing on T lymphocyte responses and optimizing for human seasonal influenza viruses (H1N1, H3N2, Victoria, and Yamagata). Designed HA-Mosaic antigens containing the most promising cellular epitopes for seasonal influenza viruses may provide broad protection against influenza.

## Materials and methods

### Influenza virus genome sequences collection

HA sequences of all human H1N1, H3N2, Victoria, and Yamagata from 2009 to 2021 used in this study were collected from Global Initiative on Sharing All Influenza Data (GISAID) and the National Center for Biotechnology Information (NCBI), including 55,514 sequences of H1, 74,410 sequences of H3, 19,197 sequences of HA-Victoria, 16,614 sequences of HA-Yamagata.

### Mosaic antigen design and optimization

**Figure 2:**
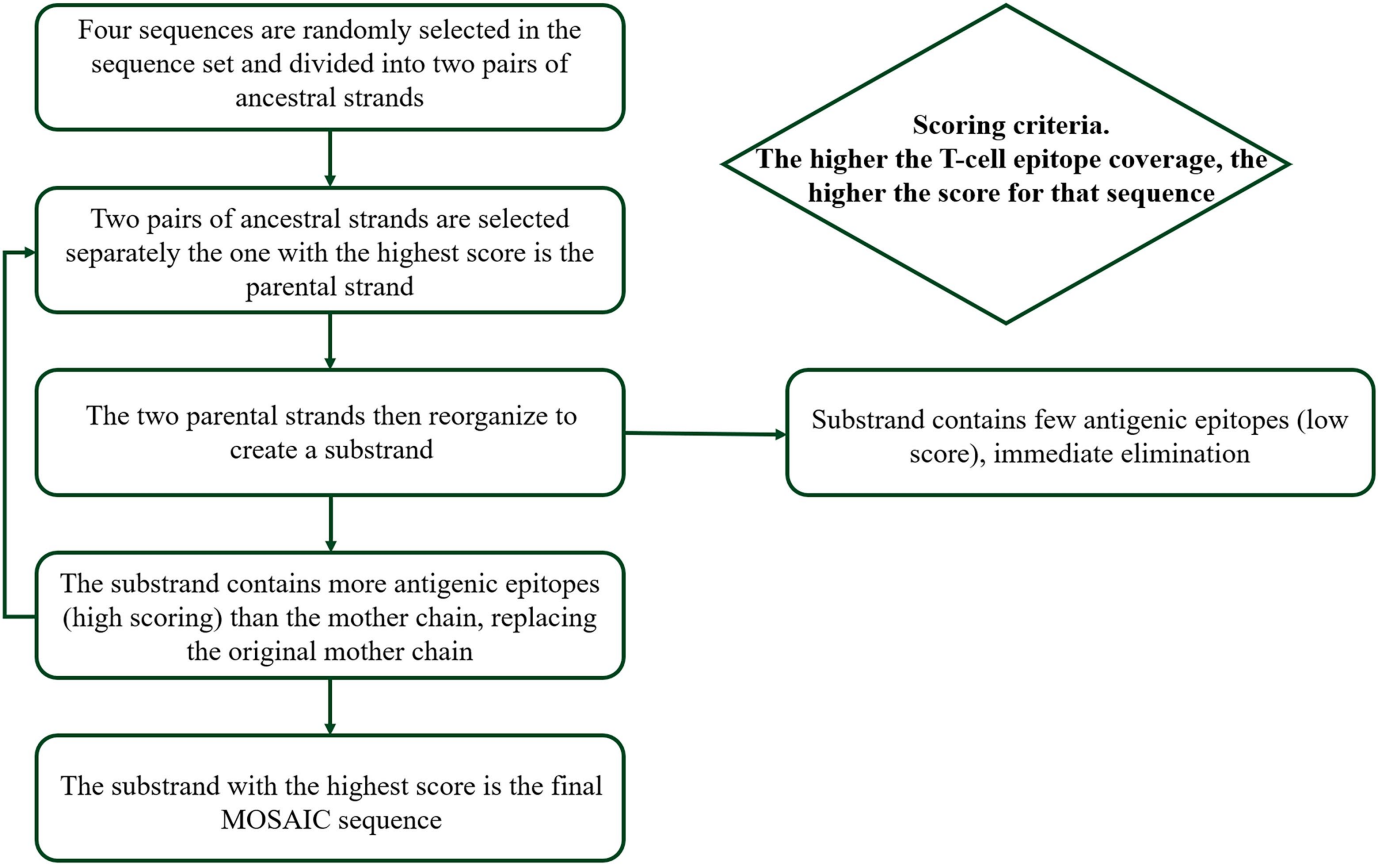
Design of mosaic antigen as a potential universal influenza vaccine based on conserved CTL epitopes. The genetic algorithm was used to optimize each population successively to generate new recombinants, and the epitope coverage was calculated and tested.

**Figure 3:**
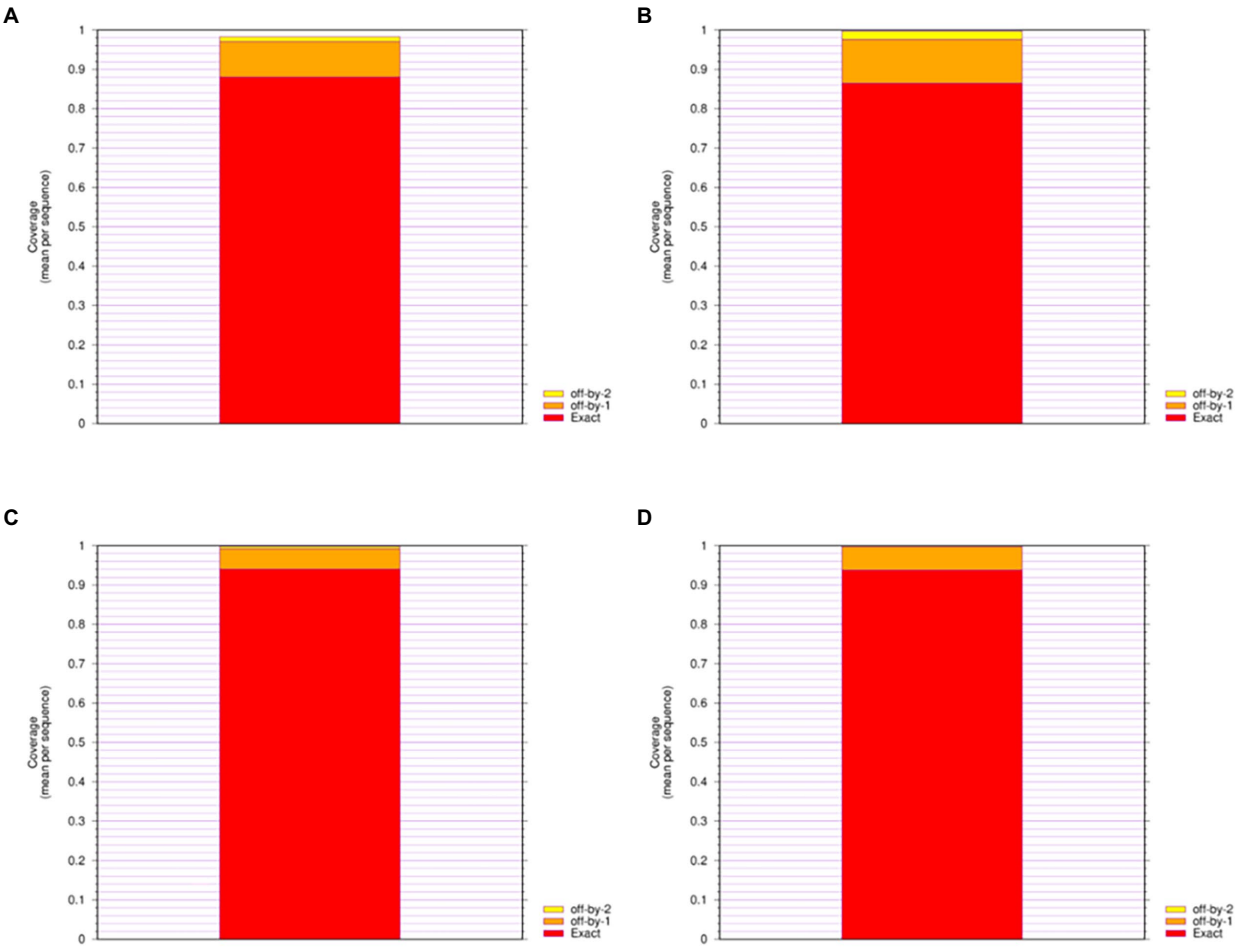
Epitope Coverage of Mosaic recombinant antigen. The results indicated coverage of **(A)** H1m-H1; **(B)** H1m-H3; **(C)** H1m-Vic; **(D)** H1m-Yam. The mean epitope coverage over the HA amino acid sequences of seasonal influenza virus vaccine strains from 2009 to 2021 was covered by the CTL epitope in mosaic recombinant antigen. “Exact” indicates up to 9/9 match; “off-by-1” indicates up to 8/9 match; and “off-by-2” indicates up to 7/9 match.

We finally obtained 7,609 amino acid sequences of H1, 9,262 amino acid sequences of H3, 3,206 amino acid sequences of HA-Victoria, and 2,198 amino acid sequences of HA-Yamagata, with optimization to avoid redundancy in the dataset by using software to remove repetitive sequences and low-quality sequences and laboratory strains based on annotation information from NCBI. Then, a genetic algorithm was used to optimize each population in turn, in which new recombinants were generated and their epitope coverage was calculated and tested (Figure 2). The basic principle of Mosaic antigen design strategy is to maximize coverage of potential T-cell epitopes found in natural circulating strains (Fischer et al., 2007). The processed amino acid sequences were uploaded to the Mosaic Vaccine Designer in FAS format, and the following parameters were set: Cocktail Size was set to “1” to obtain 1 Mosaic sequence for further use; the epitope length was set to “9” to obtain Mosaic sequences covering more CD8+ CTL cell epitopes. The threshold value was set to “3” to reduce rare epitopes that occur less frequently in natural epitopes; the maximum run time was 100 h, the population size was 500, the cycle time was 10 and the iteration value was 0 to iterate to the stagnation factor. After a genetic algorithm, a series of Mosaic sequences consisting of nine amino acids was obtained. Finally, four Mosaic recombinant antigen sequences were obtained (Figure 3).

### Identification of Mosaic recombinant antigen sequences

#### Epicover and posicover

Epitope Coverage of Mosaic recombinant antigen was evaluated using Epitope Coverage Assessment Tool (Epicover). The Mosaic recombinant antigen sequence was first added to the corresponding site as an antigen protein and was used previously in GISAID and NCBI Download analysis and then a good full strain protein amino acid sequence set to test set was added to the corresponding position Set the table a length of “9,” the maximum amino acid mismatches to score at “2,” and the minimum number of occurrences of a potential epitope in viral protein set to “3” to consider for coverage. To calculate the final result of the Mosaic of recombinant antigen sequences of all background proteins which was expressed by the average of a coverage set table.

Positional Epitope Coverage Assessment Tool (Posicover) was used to analyze the epitope coverage over the position of Mosaic recombinant antigen in each amino acid. First, Mosaic recombinant antigen was added to the corresponding position as an antigen protein. Meanwhile, the amino acid sequence of the strain downloaded and analyzed from GISAID and NCBI was set as the test protein set and added to the corresponding position. Set nominal epitope length at “9,” set antigen counts to compute upper bounds at “3,4,” and use the default values for other parameters. The final result is expressed as average table bit coverage.

#### Phylogenetic analysis

The HA amino acid sequences of seasonal influenza virus vaccine strains from 2009 to 2022 were obtained from GISAID and analyzed by sequence alignment of the Mosaic recombinant antigen sequences using MAFFT software. Representative strains of each HA sequence were compared using ClustalW and a maximum likelihood phylogenetic tree was constructed using MEGA7.

#### Tertiary structure prediction

The natural immune function of Mosaic recombinant antigen was evaluated by simulating the three-dimensional (3D) structure model of Mosaic recombinant antigen. The sequences of mosaic recombinant antigen were submitted to the SWISS-MODEL and then the homology-derived conformational model was generated with 7KNA (from A/Michigan/45/2015) as the template for HAm-H1, 4WE8 (from A/Victoria/361/2011) as the template for HAm-H3, 5YKC (from A/chicken/Taiwan/0502/2012) as the template for HAm-Vic and HAm-yam proteins. The quality of the structure was evaluated by using the Global Model Quality Estimate (GMQE) method. We further used Robetta to predict the monomeric structure of the mosaic recombinant antigen, a protein structure prediction service continually evaluated through CAMEO.

## Results

### Design of the Mosaic antigens based on potential T-cell epitopes

Mosaic recombinant antigens have shown better features for the development of vaccines against highly variable pathogens, including HIV and influenza (Barouch et al., 2010; Kamlangdee et al., 2016). The basic principle of Mosaic antigen design is to maximize coverage of potential T-cell epitopes (Th and CTL epitopes) found in circulating strains. Using the genetic algorithm of the Mosaic vaccine design strategy, we analyzed the HA amino acid sequences of seasonal influenza virus vaccine strains from 2009 to 2021, with a focus on T lymphocyte responses. The Mosaic recombinant antigen consists of nine amino acids (common length of CTL epitopes) that are identified in a large number of different natural sequences. Finally, we obtained four different Mosaic sequences of the HA amino acid sequences of H1N1, H3N2, Victoria, and Yamagata, named HAm-H1, HAm-H3, HAm-Vic, and HAm-Yam, as a cocktail combination of seasonal influenza Mosaic HA antigens (Supplementary Data).

### Screening and identification of Mosaic recombinant antigen sequences

The Mosaic recombinant antigen sequences were analyzed by epitope coverage analysis, Phylogenetic analysis, and spatial conformation analysis.

#### A significant number of potential T-cell epitopes of Mosaic recombinant antigens

We give four different Mosaic sequences and a test set of background proteins (HA amino acid sequences of seasonal influenza virus vaccine strains from 2009 to 2021) and calculate the proportion of peptides of all epitope lengths in the test sequence set that match peptides of nine amino acid epitope lengths in the Mosaic recombinant antigen. The results are expressed as the average epitope coverage of the tested sequences. The average coverage of the overall epitopes by the Mosaic recombinant antigen sequences is shown in Figure 3 and Table 1. In each Mosaic sequence, over 81.82% of the CTL epitopes matched exactly 9 amino acids on the natural protein sequence of the influenza virus (9/9 matches). In addition, the epitope coverage of incomplete amino acid epitopes (8/9 or 7/9 matches) was as high as 96.81%–99.99%, and these incomplete CTL epitopes would also function under most conditions. The results showed that the CTL epitopes in the mosaic cocktail matched well with their counterparts in circulating seasonal influenza viruses. As shown in Table 2, the CTL epitopes of the four mosaic recombinant antigens were higher or equal to the predicted epitopes in the natural HA amino acid sequences of the vaccine strains, using seasonal influenza viruses that have been in circulation for the past 5 years.

**Table 1:** The mean epitope coverage overall test sequences covered by the CTL epitope in testing sequences in mosaic sequences.

Mosaic antigen	Off-by-0	Off-by-1	Off-by-2
HAm-H1	88.04%	97.09%	98.30%
HAm-H3	86.46%	97.60%	99.69%
HAm-Vic	94.02%	99.20%	99.78%
HAm-Yam	93.79%	99.75%	99.99%

**Table 2:** The number of potential cytotoxic T lymphocyte (CTL) epitopes in mosaic antigen and seasonal influenza virus HA sequences.

Antigen	HLA-A	HLA-B	Antigen	HLA-A	HLA-B
HAm-H1	57	80	HAm-H3	62	70
A/Victoria/2570/2019 (H1N1)	54	76	A/Cambodia/E0826360/2020 (H3N2)	60	64
A/Guangdong-Maonan/SWL1536/2019 (H1N1)	54	77	A/Hong Kong/2671/2019 (H3N2)	62	69
A/Brisbane/02/2018 (H1N1)	47	77	A/Kansas/14/2017 (H3N2)	60	63
HAm-Vic	54	49	HAm-Yam	56	53
B/Washington/02/2019 (Victory lineage)	55	50	B/Phuket/3073/2013 (Yamagata lineage)	57	53
B/Colorado/06/2017 (Victory lineage)	54	50			

**Figure 4:**
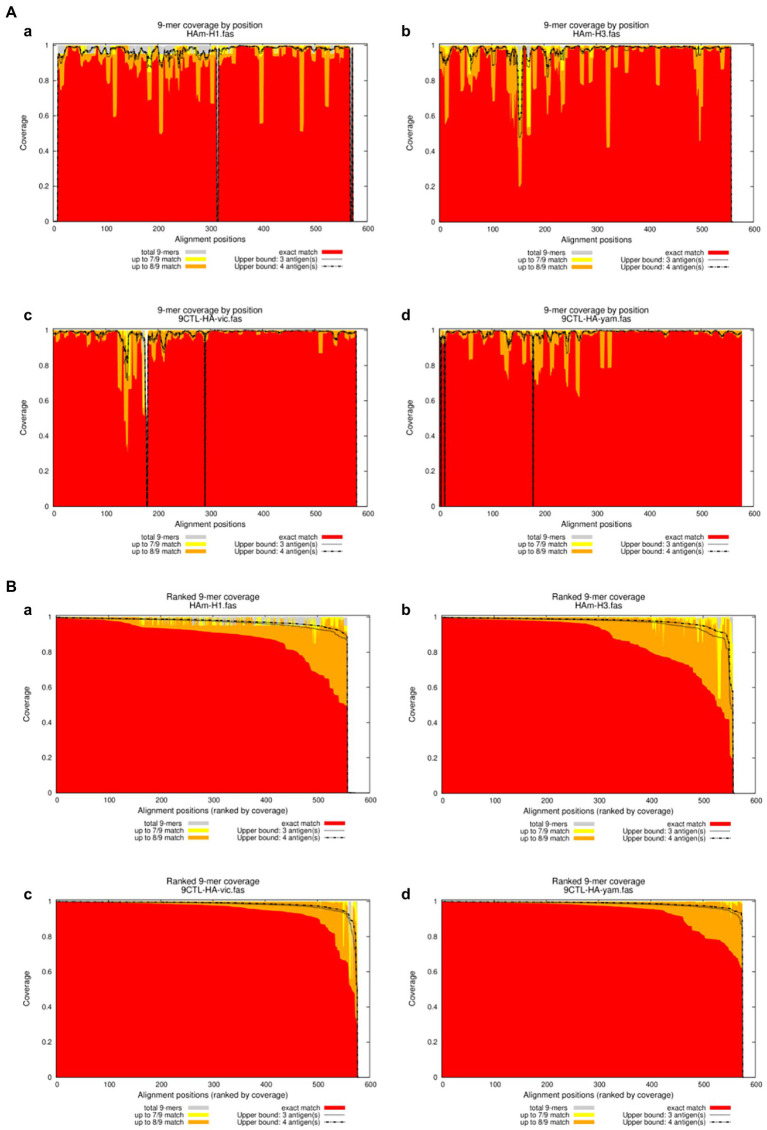
Positional Epitope Coverage of Mosaic recombinant antigen. **(A)** 9-mer coverage by position. **(B)** Ranked 9-mer coverage. The results indicated epitope coverage over the position of **(a)** H1m-H1; **(b)** H1m-H3; **(c)** H1m-Vic; and **(d)** H1m-Yam. Matched 9-mers compared to upper bounds (ranked): Proportion of 9-mers in the test set “the HA amino acid sequences of seasonal influenza virus vaccine strains from 2009 to 2021” that is exactly and approximately matched in the antigen/peptide set “Mosaic recombinant antigen,” based on a sliding window across the alignment. Each x-value represents a column in the multiple sequence alignment, ranked from left to right by exact-match proportion (descending); the *y*-values represent proportions of the background sequences at that position which have a 9-mer that is matched at different levels in the sequences in the antigen/peptide set. Exact match proportions are shown in red, 1-off (8/9) in orange and 2-off (7/9) in yellow.

We further calculated the proportion of all epitope long peptides matching the 9 amino acid epitope long peptide in each recombinant antigen from a set of HA amino acid sequences of seasonal influenza virus vaccine strains from 2009 to 2021. The results are expressed as epitope coverage at the position of the tested sequences. As shown in Figure 4, the results show that the CTL epitopes in each mosaic antigen matched well with their corresponding epitopes, with high epitope coverage for each amino acid and low deletion rates for the nine amino acids overall. In addition, we simultaneously predicted the number of CTL antigen epitopes and Th antigen epitopes in the mosaic recombinant sequence compared with the influenza vaccine strain sequence and found that although the mosaic antigen designed for CTL epitopes was selected for the vaccine design strategy, the number of Th epitopes was also significant, as shown in Table 3. This suggests that mosaic antigens may stimulate both CD4+ and CD8+ T cells and stimulate humoral and cellular immunity.

**Table 3:** The number of potential T helper lymphocyte (Th) epitopes in mosaic antigen and seasonal influenza virus HA sequences.

Antigen	HLA-A	HLA-B	Antigen	HLA-A	HLA-B
HAm-H1	58	45	HAm-H3	55	53
A/Victoria/2570/2019 (H1N1)	59	44	A/Cambodia/E0826360/2020 (H3N2)	55	53
A/Guangdong-Maonan/SWL1536/2019 (H1N1)	61	45	A/Hong Kong/2671/2019 (H3N2)	61	54
A/Brisbane/02/2018 (H1N1)	60	45	A/Kansas/14/2017 (H3N2)	48	50
HAm-Vic	47	55	HAm-Yam	58	57
B/Washington/02/2019 (Victory lineage)	44	57	B/Phuket/3073/2013 (Yamagata lineage)	60	58
B/Colorado/06/2017 (Victory lineage)	45	55			

#### Mosaic recombinant antigens are closely related to influenza vaccine strains

**Figure 5:**
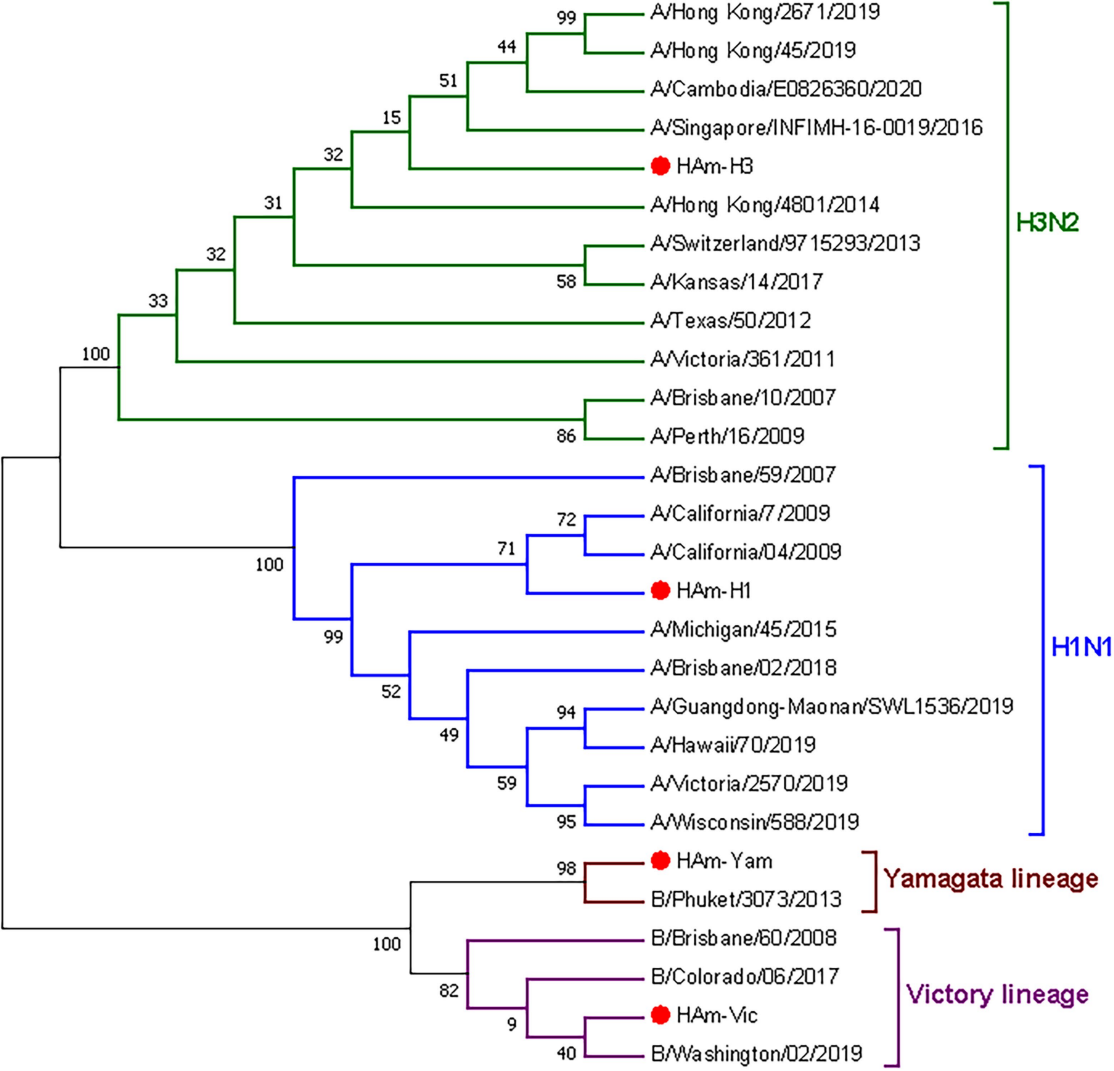
Phylogenetic relationship of the seasonal influenza virus vaccine strains hemagglutinin proteins and mosaic recombinant antigens. Four mosaic recombinant antigens (HAm-H1, HAm-H3, HAm-Vic, and HAm-Yam) are marked by red dots. H1N1 (blue), H3N2 (green), Victory lineage (violet), and Yamagata lineage (brown) phylogenic groups are indicated. Representative strains for each HA subtype were aligned using ClustalW alignment and a maximum-likelihood phylogenetic tree was constructed using MEGA7.

We proceeded to analyze the genetic evolution of the mosaic HA gene and the natural HA gene of seasonal influenza virus vaccine strains from 2009 to 2022. The phylogenetic tree of the HA gene is shown in Figure 5. The results showed a close genetic relationship between the mosaic recombinant antigen and the vaccine strains for each year, indicating that the mosaic recombinant antigen involved in this study has sufficient potential as a vaccine antigen.

#### Mosaic recombinant antigens have a monomeric and trimeric structure similar to that of the natural HA protein

**Figure 6:**
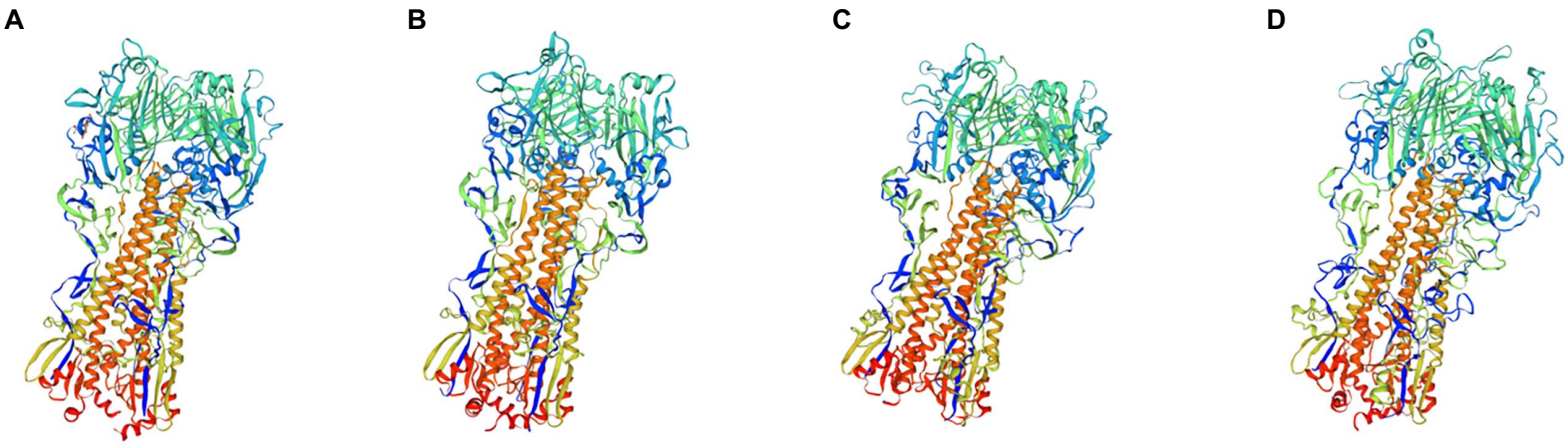
The three-dimensional (3D) structure model of Mosaic recombinant antigen. The results indicated a 3D structure model of **(A)** H1m-H1; **(B)** H1m-H3; **(C)** H1m-Vic; and **(D)** H1m-Yam. Mosaic recombinant antigen sequences were submitted to the SWISS-MODEL server to construct the homology-derived conformational model and then evaluated its quality with the global model quality estimate (GMQE) method.

**Figure 7:**
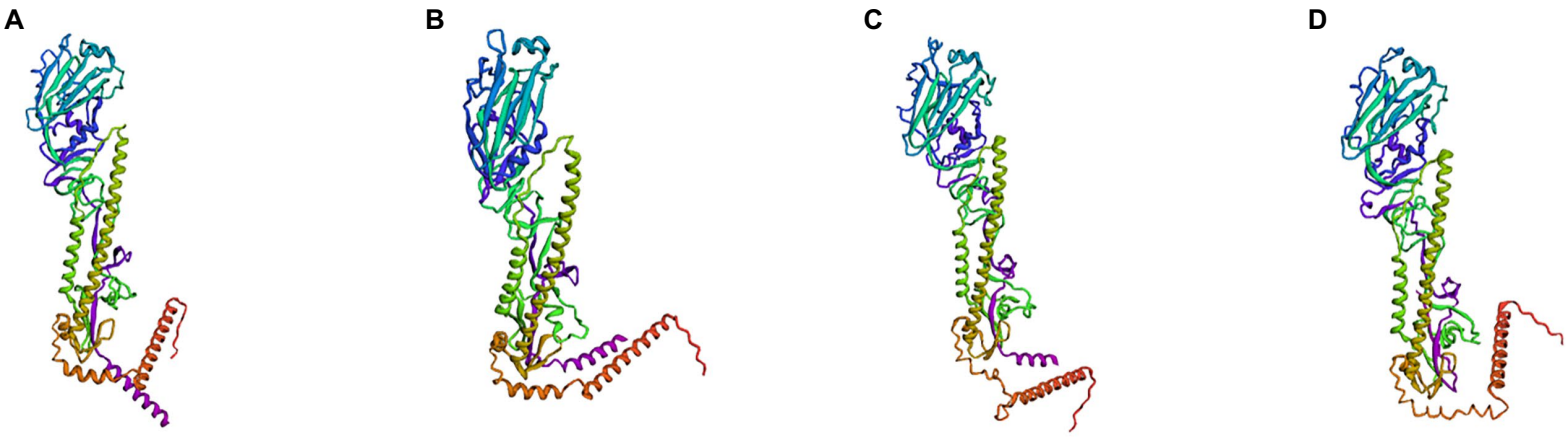
The three-dimensional (3D) structure model of monomer to Mosaic recombinant antigen. The results indicated a 3D structure model of monomer to **(A)** H1m-H1; **(B)** H1m-H3; **(C)** H1m-Vic; and **(D)** H1m-Yam. Mosaic recombinant antigen sequences were submitted to the Robetta server to construct the homology-derived conformational model.

Subsequently, we simulated and predicted the conformation of the recombinant mosaic antigens utilizing a three-dimensional structural model to compare their similarity to the prevalent seasonal influenza virus, and then assessed whether the recombinant mosaic antigens could be expressed and purified as immunogens to further their role in immune function (Figures 6, 7). The global model quality estimates (GMQE) scores for the four mosaic recombinant antigens and their template proteins were as follows: HAm-H1 protein: 0.74, HAm-H3 protein: 0.79; HAm-Vic protein: 0.49, and HAm-Yam protein: 0.49. Since the IBV strains in the sample pool predicted by SWISS-MODEL are monomers, the system recommended strain A/chicken/Taiwan/0502/2012 was used to predict the trimeric structure of HAm-Vic and HAm-yam, but its sequence similarity was only about 30%, resulting in a low GMQE score (GMQE is related to the coverage rate of, i.e., a model covering only half of the target sequence is unlikely to get a score above 0.5.). Therefore, we further used Robetta to investigate the monomeric structure of the Mosaic recombinant antigen. It consists of three non-covalently bound HA protein monomers, and the monomer structure is informative for the complete 3D structure of the HA protein. The confidence for the four mosaic recombinant antigens was: HAm-H1 protein: 0.77, HAm-H3 protein: 0.76; HAm-Vic protein: 0.61, HAm-Yam protein: 0.58 (1.0 good, 0.0 bad. Confidence levels correspond to the average paired TM scores of the top 10 Rosetta scoring models. These metrics are considered to correlate with the actual GDT to the original.).

The results showed that the mosaic recombinant antigen had a high structural similarity to the natural protein.

## Discussion

Influenza virus antigens are constantly evolving and frequently drifting, leading to annual outbreaks and occasional pandemics (Keshavarz et al., 2019). Given the current situation, the development of an influenza vaccine that induces a broad-spectrum durable immune response as a universal influenza vaccine is an important direction for new influenza vaccine research and development. The National Institute of Allergy and Infectious Diseases (NIAID) convened a workshop on universal influenza vaccines in 2017 and set a specific goal for the development of a universal influenza vaccine: to provide at least 1 year of protection and 75% effectiveness in any person infected with group 1 or 2 A viruses (Erbelding et al., 2018).

Currently, all influenza vaccines available are mainly divided into inactivated influenza vaccine (IIV), live attenuated influenza vaccine (LAIV), and recombinant influenza vaccine (RIV). These vaccine strategies most induce specific neutralizing antibodies against specific HA and exert humoral immunity, but nearly do not induce CTL and only weakly enhance the existing memory CD8+ T-cell response. The long-term protective value of any vaccine against specific hemagglutinin or neuraminidase will inevitably diminish over time as influenza viruses are under constant selective pressure. Immune CD8+ T cells are essential for recovery and provide some protection against severe influenza disease, including that caused by avian influenza virus infections never before encountered (Auladell et al., 2019).

CD8+ T-cell responses are also known to play a decisive role in controlling primary influenza virus infection (Brincks et al., 2008), reducing viral load, and protecting the respiratory tract (Brown and Kelso, 2009; Weinfurter et al., 2011). In mice, CTLs targeting conserved epitopes contribute to protective immunity against various subtypes of influenza viruses (Kuwano et al., 1988). In this study, we focused on antigen design, using the Mosaic vaccine design strategy and genetic algorithms to design a set of multivalent vaccine antigens that focused on T-lymphocyte responses and were optimized for human seasonal influenza viruses (H1N1, H3N2, Victoria, and Yamagata). A cocktail of mosaic HA sequences containing the most potential CTL epitopes for seasonal influenza viruses was designed.

In recent years, a growing number of studies have identified a critical role for cytotoxic T lymphocytes (CTL) in anti-influenza immunity and clearance of influenza viruses (Rimmelzwaan et al., 2009; Cargnelutti et al., 2013; Auladell et al., 2019). Over the years, it has been found that the cross-protective immunity generated by alloimmune vaccines, such that all influenza A viruses, regardless of subtype or strain, induce immunity to some extent, is mediated primarily by CD8+ cytotoxic T lymphocytes (CTL), which recognize conserved internal components of the virus (Lee et al., 2008; Reber et al., 2018). Sherritt et al. (2000) have used peptide vaccines in the preexisting multiple CD8+ T-cell epitopes delivered in mice with CD8+ T-cells specific for one of these epitopes and showed that it induced CTL with additional specificity. In addition to data from animals supporting a role for CD8+ T-cell responses in viral clearance and survival (Talker et al., 2016; McMahon et al., 2019), there are also relevant data showing the presence of cross-reactive influenza virus-specific CD8+ memory T cells in humans (Boon et al., 2004; Kreijtz et al., 2008; Duan and Thomas, 2016; Grant et al., 2016). A separate study by McMichael et al. (1983) showed that high levels of CD8+ T cells were associated with reduced viral shedding following experimental infection in individuals lacking specific antibodies.

A CD8+ T-cell-induced vaccine needs to contain a sufficient number of conserved epitopes to cover the different MHC class I haplotypes of an individual. In the present study, the number of potential CTL epitopes for the Mosaic recombinant antigen was higher or equal to that of recent seasonal influenza vaccine strains, and the results are shown in Table 2. Experimentally validated T-cell epitopes reported in the Immune Epitope Database (IEDB) are also included in the predicted epitopes for the Mosaic antigen, such as GLFGAIAGFI (Gianfrani et al., 2000; Toebes et al., 2006; Kosor Krnic et al., 2008; Alexander et al., 2010), YYSTAASSL (Muralidharan et al., 2018; Hensen et al., 2021), GLDNHTILL (Koutsakos et al., 2019), LYGDSKPQKF (Hensen et al., 2021), YVKQSTLKLA (Markovic-Plese et al., 2005), MKAILVVLLYTFATAN (Herrera et al., 2013), SLNDDGLDNHTILL (Koutsakos et al., 2019), and RFEIFPKTSSWPNHDSNKG (Herrera et al., 2013) which have been reported in previous studies. Although some mutations that evade influenza virus-specific CTL recognition have been reported, many epitopes have remained unchanged throughout the evolution of influenza viruses, suggesting that functional limitations in these regions create unsustainable fitness costs when mutated (Voeten et al., 2000). As influenza is an acute infection that can eventually be cleared by the production of an antibody response, CTL escape mutations have little impact on individuals (Goulder et al., 1997). Th epitopes are mainly presented by MHC class II molecules, and Th cells can stimulate B cells to produce antibodies and activate CTL; thus, when Th epitopes synergize with CTL epitopes, the protective effect can be significantly enhanced. Based on this theoretical basis, we also predicted a comparison of the number of Th antigen epitopes in the mosaic recombinant sequence with that of the influenza vaccine strain and showed that the number of potential Th epitopes in the mosaic recombinant antigen was higher than or equal to that of the recent seasonal influenza vaccine strain (Table 3). This suggests that the mosaic antigen has the potential to stimulate both CD4+ and CD8+ T cells and stimulate both humoral and cellular immunity in the body, which is a previously unanticipated surprise. Multiple studies have also shown that co-induction of B-cell and T-cell immunity is beneficial because virus-specific T cells often cross-react to different IAV strains (Boon et al., 2004; Sridhar, 2016; Cookenham et al., 2020).

Since early exposure of the population to influenza viruses results in the immune system developing the ability to respond to future exposures, i.e., preexisting immunity, which allows the susceptible population to be exposed to a new pandemic strain without prior antibodies, the body will preferentially evoke memory responses against conserved regions rather than activating them from scratch, and thus T-cell activation vaccines may be most effective in that situation. Models of influenza virus evolution, although imperfect, have shown that transient strain-spanning immunity, possibly biologically mediated by heterotypic T-cell responses to conserved antigenic epitopes, is critical to explaining the limitations of viral diversity in host populations (Ferguson et al., 2003; Brown and Kelso, 2009). With the widespread use of the influenza vaccine, improved vaccines that additionally induce cross-protective responses may help to avoid the theoretical increase in strain diversity and the corresponding increase in the difficulty of disease control. If vaccines that significantly boost heterosubtypic CD8+ T-cell responses in addition to inducing antibodies to circulate strains were in general use, the general population would have some protection at the onset of a pandemic. Despite the inappropriate specificity of antibodies, CD8+ T-cell responses have the potential to reduce the morbidity and mortality normally associated with emerging virus subtypes.

Our study has its limitations. The *in vivo* immunogenicity and protective efficacy of the Mosaic antigen cocktail vaccine against seasonal influenza virus is in the process of further validation. In this study, modifying an existing neutralizing antibody-based seasonal influenza virus vaccine to include a component that activates cross-protective T cells would provide an attractive strategy for improving human protection against seasonal influenza virus drift and mutation while reducing the impact of future pandemic strains. Our study provides an idea for the development of a rationally designed influenza vaccine targeting T lymphocyte immunity.

## Data availability statement

The original contributions presented in the study are included in the article/Supplementary material, further inquiries can be directed to the corresponding author.

## Author contributions

YS contributed to the conceptualization and editing of the manuscript. YS and CS contributed to the resources and supervision. XL and ML performed the methodology. LW performed the experiments. XL and TZ wrote the manuscript. TZ proofread the final version. All authors contributed to the article and approved the submitted version.

## Funding

This research was funded by the National Key Research and Development Program of China (grant number: 2021YFC2300100), the National Natural Science Foundation of China (grant number: 82041043), and Shenzhen Science and Technology Program (grant numbers: KQTD20180411143323605, JSGG20200225152008136, GXWD20201231165807008, and 20200825113322001).

## Conflict of interest

The authors declare that the research was conducted in the absence of any commercial or financial relationships that could be construed as a potential conflict of interest.

## Publisher’s note

All claims expressed in this article are solely those of the authors and do not necessarily represent those of their affiliated organizations, or those of the publisher, the editors and the reviewers. Any product that may be evaluated in this article, or claim that may be made by its manufacturer, is not guaranteed or endorsed by the publisher.
