# Supplementary material for: Strategies targeting hemagglutinin cocktail as a potential universal influenza vaccine
Source: Front Microbiol. 2022 Sep 29;13:1014122. doi: 10.3389/fmicb.2022.1014122 (PMC9558277; doi:10.3389/fmicb.2022.1014122)
Supplement: Supplementary file 1 [file Data_Sheet_1.ZIP › 9.9-new-Supplementary Materials/Supplementary Data 1-The HA mosaic sequence.docx]

**The HA mosaic sequence**

>HAm-H1

MKQGKTKATKMKAILVVLLYTFATANADTLCIGYHANNSTDTVDTVLEKNVTVTHSVNLLEDKHNGKLCKLRGVAPLHLGKCNIAGWILGNPECESLSTASSWSYIVETSSSDNGTCYPGDFIDYEELREQLSSVSSFERFEIFPKTSSWPNHDSNKGVTAACPHAGAKSFYKNLIWLVKKGNSYPKLSKSYINDKGKEVLVLWGIHHPSTSADQQSLYQNADAYVFVGTSRYSKKFKPEIAIRPKVRDQEGRMNYYWTLVEPGDKITFEATGNLVVPRYAFAMERNAGSGIIISDTPVHDCNTTCQTPKGAINTSLPFQNIHPITIGKCPKYVKSTKLRLATGLRNVPSIQSRGLFGAIAGFIEGGWTGMVDGWYGYHHQNEQGSGYAADLKSTQNAIDKITNKVNSVIEKMNTQFTAVGKEFNHLEKRIENLNKKVDDGFLDIWTYNAELLVLLENERTLDYHDSNVKNLYEKVRSQLKNNAKEIGNGCFEFYHKCDNTCMESVKNGTYDYPKYSEEAKLNREEIDGVKLESTRIYQILAIYSTVASSLVLVVSLGAISFWMCSNGSLQCRICI

>HAm-H3

MKTIIALSYILCLVFAQKIPGNDNSTATLCLGHHAVPNGTIVKTITNDRIEVTNATELVQNSSIGEICDSPHQILDGENCTLIDALLGDPQCDGFQNKKWDLFVERSKAYSNCYPYDVPDYASLRSLVASSGTLEFNNESFNWTGVTQNGTSSACIRRSSSSFFSRLNWLTHLNYTYPALNVTMPNNEQFDKLYIWGVHHPGTDKDQIFLYAQSSGRITVSTKRSQQAVIPNIGSRPRIRDIPSRISIYWTIVKPGDILLINSTGNLIAPRGYFKIRSGKSSIMRSDAPIGKCKSECITPNGSIPNDKPFQNVNRITYGACPRYVKQSTLKLATGMRNVPEKQTRGIFGAIAGFIENGWEGMVDGWYGFRHQNSEGRGQAADLKSTQAAIDQINGKLNRLIGKTNEKFHQIEKEFSEVEGRIQDLEKYVEDTKIDLWSYNAELLVALENQHTIDLTDSEMNKLFEKTKKQLRENAEDMGNGCFKIYHKCDNACIGSIRNGTYDHNVYRDEALNNRFQIKGVELKSGYKDWILWISFAISCFLLCVALLGFIMWACQKGNIRCNICI

>HAm-Vic

MKAIIVLLMVVTSNADRICTGITSSNSPHVVKTATQGEVNVTGVIPLTTTPTKSHFANLKGTETRGKLCPKCLNCTDLDVALGRPKCTGKIPSARVSILHEVRPVTSGCFPIMHDRTKIRQLPNLLRGYEHVRLSTHNVINAEDAPGGPYKIGTSGSCPNITNGNGFFATMAWAVPKNDKNKTATNPLTIEVPYICTEGEDQITVWGFHSDNETQMAKLYGDSKPQKFTSSANGVTTHYVSQIGGFPNQTEDGGLPQSGRIVVDYMVQKSGKTGTITYQRGILLPQKVWCASGRSKVIKGSLPLIGEADCLHEKYGGLNKSKPYYTGEHAKAIGNCPIWVKTPLKLANGTKYRPPAKLLKERGFFGAIAGFLEGGWEGMIAGWHGYTSHGAHGVAVAADLKSTQEAINKITKNLNSLSELEVKNLQRLSGAMDELHNEILELDEKVDDLRADTISSQIELAVLLSNEGIINSEDEHLLALERKLKKMLGPSAVEIGNGCFETKHKCNQTCLDRIAAGTFDAGEFSLPTFDSLNITAASLNDDGLDNHTILLYYSTAASSLAVTLMIAIFVVYMVSRDNVSCSICL

>HAm-Yam

MKAIIVLLMVVTSNADRICTGITSSNSPHVVKTATQGEVNVTGVIPLTTTPTKSYFANLKGTRTRGKLCPDCLNCTDLDVALGRPMCVGTTPSAKASILHEVRPVTSGCFPIMHDRTKIRQLPNLLRGYEKIRLSTQNVIDAEKAPGGPYRLGTSGSCPNATSKIGFFATMAWAVPKDNYKNATNPQTVEVPYICTEGEDQITVWGFHSDNKTQMKSLYGDSNPQKFTSSANGVTTHYVSQIGDFPDQTEDGGLPQSGRIVVDYMVQKPGKTGTIVYQRGVLLPQKVWCASGRSKVIKGSLPLIGEADCLHEEYGGLNKSKPYYTGKHAKAIGNCPIWVKTPLKLANGTKYRPPAKLLKERGFFGAIAGFLEGGWEGMIAGWHGYTSHGAHGVAVAADLKSTQEAINKITKNLNSLSELEVKNLQRLSGAMDELHNEILELDEKVDDLRADTISSQIELAVLLSNEGIINSEDEHLLALERKLKKMLGPSAVDIGNGCFETKHKCNQTCLDRIAAGTFNAGEFSLPTFDSLNITAASLNDDGLDNHTILLYYSTAASSLAVTLMLAIFIVYMVSRDNVSCSICL
